# Preclinical biodistribution and safety evaluation of human iPSC-derived dopaminergic neural progenitor cells for Parkinson’s disease

**DOI:** 10.3389/fcell.2025.1701748

**Published:** 2026-01-02

**Authors:** Ying Huang, Hairuo Wen, Lily Li, Lulu Li, Qianqian Li, Chao Qin, Yiyang Mao, Zhi Lin, Hua Jiang, Frank Zhu, Xiang Li, Xingchao Geng

**Affiliations:** 1 Institute of Safety Evaluation, National Institutes for Food and Drug Control, Beijing, China; 2 Beijing Key Laboratory of Quality Control and Nonclinical Research and Evaluation for Cellular and Gene Therapy Medicinal Products, Beijing, China; 3 XellSmart Biopharmaceutical (Suzhou) Co., Ltd, Suzhou, China

**Keywords:** biodistribution, human iPSC-derived dopaminergic neural progenitor cells, in vivo differentiation, Parkinson’s disease, safety evaluation study

## Abstract

**Introduction:**

Human pluripotent stem cells (PSCs) have the potential to revolutionize regenerative medicine, but their clinical safety has not been thoroughly investigated. We investigated the *in vivo* biodistribution, safety evaluation, and *in situ* tumorigenicity test of specific human iPSC-derived dopaminergic neural precursor cell (DAP) therapeutic products in a severe immunodeficient mouse model and established a method for detecting stereotactic drug delivery and distribution differentiation to support clinical trial dose justification and toxicity monitoring.

**Methods:**

For the biodistribution study, DAPs were injected into the unilateral striatum of NSG mice, and the distribution and differentiation of the transplanted cells were determined via immunofluorescence staining and qPCR at 1-, 28-, 84-, and 168-days post-administration. The toxicity and tumorigenicity studies were carried out on NSG mice by administering saline, 1 × 10^5^ DAP cells, 2 × 10^5^ DAP cells, 0.01% iPSCs (2 × 10^5^ cells) or 1% iPSCs (2 × 10^5^ cells) per animal in accordance with the intended clinical dosage. After 28, 84, and 168 days, the mice were euthanized.

**Results:**

Brain-only discovery of DAP markers (Ki67, FOXA2, OTX2, STEM101, and STEM121) and specific sequences of DAPs was confirmed. From 1- to 184-days, the copy number of *Th* first decreased but then increased; the expression of STEM121 decreased, and the neuronal cell marker proteins Th and STEM101 increased. Additionally, the differentiation target RNA *Th* was identified 28 days after administration, and both the differentiation ratio and degree increased. There was no evidence of toxicity from DAPs, and there were no tumors or abnormally proliferating cells detected.

**Discussion:**

This study developed a novel method for determining biodistribution and differentiation *in vivo*, provided a strategy to evaluate the safety of iPSC derived DAPs, and showed their safety in mice. The data provides essential safety data for the clinical translation of DAPs and supports their phase I clinical trials in China and the United States.

## Introduction

Human induced pluripotent stem cells (hiPSCs), which are independent of the donor, are commonly obtained by the reprogramming of peripheral blood mononuclear cells, fibroblasts, or mesenchymal stromal cells from healthy donors or patients (Park et al., 2008; Takahashi et al., 2007). Autologous transplantation for patients could be made possible, and specific types of cells could be induced and enriched using differentiation methods (Stoddard-Bennett and Pera, 2020; Stoddard-Bennett and Reijo Pera, 2019). Therefore, hiPSCs are capable of overcoming the challenges of excessive immune response, production and availability restrictions, and ethical and legal controversies (Nakamura et al., 2023). HiPSCs are now viewed as a highly promising therapeutic strategy for rare and difficult-to-treat diseases, including Leigh syndrome (Sequiera et al., 2022) and bullous keratopathy (Hatou et al., 2021). Approximately 1%–2% of people over the age of 65 have Parkinson’s disease (PD), which is a progressive movement disorder of the nervous system. Due to the unclear pathogenic mechanism, there is currently a lack of effective treatment methods. The loss of midbrain neurons and reduced dopamine levels in the striatum are the major manifestations of PD, which subsequently results in motor disorders (Liu and Cheung, 2020; Pires et al., 2017; Kirkeby et al., 2023). Moreover, new hope for PD patients is being offered by iPSCs that are differentiated from dopaminergic neural progenitor cells (DA NPCs) (Cha et al., 2023; Mominur et al., 2022).

Reprogrammed human somatic cells are used to derive iPSCs for PD patients, and the production and quality control processes are complex. Safety risks, such as tumorigenicity, ectopic distribution, and immune rejection (Mehler et al., 2020; Zhao et al., 2015), remain primary concerns before clinical translation (Myriam et al., 2023; Sonntag et al., 2018). Obtaining sufficient safety data for such products during the preclinical evaluation stage is crucial. In this study, the biodistribution and toxicity risk of a novel hiPSC-derived dopaminergic progenitor cell (DAP) for the treatment of PD were comprehensively investigated. This study innovatively established a brain micro positioning technique that could fully simulate the clinical administration method based on the B-NDG immunodeficient mouse model, as well as a method for detecting the *in vivo* distribution and differentiation of human hiPSCs after cell transplantation. To reduce immune rejection and observe long-term after transplantation, severe immunodeficient NSG mice lacking mature T, B, and NK cells were employed. On this basis, the biodistribution of iPSC-derived dopaminergic progenitor cells (DAPs) as well as a single-dose toxicity study along with *in situ* administration-induced tumorigenicity testing were performed to provide crucial evidence supporting the clinical translation and registration of DAPs.

## Materials and methods

### Cells

Human iPS cell-derived dopamine precursor cells (DAPs, 5 × 10^7^ cells/mL), 0.01% iPSC DAP cell injection (DAP-iPSC-001) and 1% iPSC DAP cell injection (DAP-iPSC-100) were provided by XellSmart Biopharmaceutical (Suzhou) Co., Ltd. and were used immediately on the day of administration. The differentiation protocol used for the preparation of DAPs cells were as follows:Pluripotent stem cells (PSCs) were initially differentiated into midbrain floor plate cells in a neural induction medium composed of DMEM/F12, N2 supplement, and non-essential amino acids, supplemented with Wnt agonist and Smoothened inhibitor. The floor plate cells were treated with FGF8b and SAG to induce the floor plate cells differentiation to the midbrain dopaminergic progenitor cells (mDAPs). After differentiation, the cells were harvested and cryopreserved. The mDAPs were subsequently resolved and cultured in suspension for cell transplantation.

### Animals

SPF-grade B-NDG mice (n = 194, with equal numbers of males and females) were purchased from Biocytogen Jiangsu Gene Biotechnology Co., Ltd. (Laboratory Animal Production Nos. SCXK (Jing) 2021--0005 and SYXK (Jing) 2021--0072; animal quality certificate number: 110360231100069552). Among them, 80 mice (approximately 8 weeks old) were used for the biodistribution study, whereas 114 mice (approximately 6 weeks old) were used for the toxicological study. The experimental protocols were reviewed and approved before implementation by the Institutional Animal Care and Use Committee (IACUC) of the Institute of Safety Evaluation, National Institutes for Food and Drug Control. All animal-related operations in the study were carried out in strict accordance with the international laboratory animal ethics guidelines “Guide for the Care and Use of Laboratory Animals”. All experimental operations were conducted in a GLP laboratory with the approval of the IACUC.

### Biodistribution study of DAPs

A total of 80 B-NDG mice (40 males and 40 females) were randomized into the control group (32 animals in total) or the DAP group (48 animals in total). A single injection was administered to the left striatum of each mouse using an RWD 6803 brain stereotaxic apparatus (RWD Life Science), with 4 μL of the solvent (sodium lactate Ringer’s injection containing 0.1% vitamin C and 1% human serum albumin, provided by Sizze Biomedical (Suzhou) Co., Ltd.) or DAPs (2 × 10^5^ cells per animal)). Tissue samples were collected at 1 day, 28 days, 84 days, and 168 days post-administration. The collected tissues included the whole brain, spinal cord, heart, liver, spleen, lung, kidney, ovary and uterus (female only), testis and epididymis (male only), and blood. For each point, half of the animals in the vehicle control group and the test substance group were used for immunofluorescence staining, while the other half were used for qPCR detection.

The distribution of the DAPs in the blood, peripheral organs, and brain tissue was identified via the human cell-specific markers STEM121/STEM101, and the differentiation of the DAPs in the brain tissue was detected via human DAP markers at different stages of differentiation. STEM101 (Cat No. TAKARA, Y40400), STEM121 (Cat No. TAKARA, Y40410), TH (Cat No. AB152Millipore), Ki67 (Cat No. ab15580, Abcam), OTX2 (Cat No. BAF 1979, R&D), and FOXA2 (Cat No. AF2400, R&D) were used. The brain slices were permeabilized with 0.5% Triton X-100 for 20 min and then washed three times with PBS containing 0.1% Triton X-100 for 5 min each. The slices were blocked with serum for 1 h, followed by the addition of primary antibody (diluted in primary antibody diluent or PBS) and incubation at room temperature for 30 min and overnight at 4 °C. After rewarming at room temperature for 30 min, the slices were washed three times with PBS containing 0.1% Triton X-100 for 5 min each. The secondary antibodies used were as follows: diluted in PBS containing 0.1% Triton X-100; donkey anti-rabbit (Cat No. A21206, Invitrogen); donkey anti-mouse (Cat No. A11036, Invitrogen); goat anti-mouse (Cat No. A11030, Invitrogen); and donkey anti-goat (Cat No. A32814TR, Invitrogen). The secondary antibodies were added, and the samples were incubated at room temperature in the dark for 2 h, followed by three washes with PBS for 5 min each. Anti-fluorescence quenching mounting medium (containing DAPI) (Cat No. P0131, Beyotime Biotechnology) was added for mounting. The slices were observed and photographed under an inverted fluorescence microscope (IX71, Olympus). For peripheral organ paraffin-embedded sections, dewaxing and hydration, antigen retrieval, blocking, and addition of primary antibody were performed, followed by the same steps as those for brain tissue. Images at different magnifications were captured for each immunofluorescence-stained slide, and representative images of each marker for individual animals were selected for evaluation of the staining score.

The distribution and expression of the DAPs in the blood, peripheral organs, and brain tissue were detected via qPCR via human-specific gene sequences, and the differentiation level of the DAPs in the brain tissue was detected via identification of the dopamine neuron differentiation marker gene tyrosine hydroxylase (*Th*). The sequences of the upstream and downstream primers were “TATCGTGGAAGGACTCATGGTAT” and “AAGGAAATTATGGGAAAGCCAGTC”, respectively, and the probe sequence was “AGGCTCCCACCTTTCTCATCCAAGACTG”. All the above primers were synthesized by General Biological Co., Ltd. Fluorescence quantitative PCR (Real-Time System, CFX 96, Bio-RadRAD) was performed via a three-step method, with the following reaction conditions: pre-denaturation at 95 °C for 30 s and 40 cycles of 95 °C for 5 s and 60 °C for 30 s. The mRNAs of the animals were extracted from brain tissue samples via the TRIzol method. The Cq values of the target gene and the internal reference gene were detected via the dye method for relative quantification. The relative expression of the target mRNAs in the brain tissue of mice after the administration of the DAPs was further studied. The extracted RNA was reverse transcribed via the PrimeScript™ RT reagent Kit (Takara, RR047A) with gDNA Eraser. The prepared cDNA was subsequently used for sample detection. Fluorescence quantitative PCR was performed via a two-step method, with the following reaction conditions: pre-denaturation at 95 °C for 30 s; PCR at 95 °C for 5 s and 60 °C for 30 s for 40 cycles; and melting curve generation. qPCR data were analyzed via the use of Bio-Rad CFX Manager 3.1 software to calculate the Cq value.

### Single-dose toxicity study of DAPs

A total of 114 B-NDG mice were randomized into the control group, low-dose group (1 × 105 DAP cells/animal), high-dose group (2 × 105 DAP cells/animal), 0.01% iPSC group (2 × 105 cells/animal), and 1% iPSC group (2 × 105 cells/animal) on the basis of their body mass (Table 1). A single injection of human iPSC-derived DAPs, iPSC cell suspension or solvent was administered to the striatum of the brain. During the experiment, clinical symptoms were observed daily, while body mass and food intake were measured once per week. The mice were anesthetized with an intramuscular injection of Zoletil 50 (0.1 mL/kg). Hematological examination was performed via an ADVIA 2120i hematology analyzer (Siemens AG), and serum biochemical examination was performed via a Hitachi 7180 automated biochemical analyzer (Hitachi Limited). Organ weight and gross and histopathological examinations were performed on 28 days, 84 days and 168 days post-administration. To investigate the potential tumorigenic risk of different concentrations of iPSC residues, an *in situ* tumorigenicity examination was also included for animals in the 0.01% iPSC group and the 1% iPSC group, which were necropsied for 168 days.

**TABLE 1:** Study design of toxicity study.

Group	Test article	Dose (cells/animal)	Number of animals for Necropsy (♂/♀)		
			28d	84d	168d
Control group	Solvent	-	5/5	5/5	5/5
Low-dose group	DAPs	1 × 10^5^	5/5	5/5	5/5
High-dose group	DAPs	2 × 10^5^	5/5	5/5	5/5
0.01% iPSC group	DAPs (Containing 0.01% iPSC)	2 × 10^5^	—	—	6/6
1% iPSC group	DAPs (Containing 1% iPSC)	2 × 10^5^	—	—	6/6

### Statistical analysis

All animal body mass, food intake, body temperature, blood pressure, electrocardiogram, hematology, serum biochemistry, peripheral blood T lymphocyte subset distribution, and organ weight data are presented as the mean ± standard deviation (SD) of *n* values, where *n* corresponds to the number of mice used. Statistical analyses were performed via one-way ANOVA, followed by Dunnett’s test for comparisons with the control group. Figures were generated via GraphPad Prism 10 for Windows (GraphPad Software, San Diego, CA, United States). Statistical significance was determined via SAS (ver.12), with *p* < 0.05 considered significantly different.

## Results

### Biodistribution of DAPs in immunodeficient mice

In this study, the long-term biodistribution of DAPs in NSG mice was investigated by identifying cells expressing Ki67, STEM121, FOXA2, STEM101 and OXT2, and the sequences of the dopamine neuron-specific marker genes were recognized as DAPs via PCR at the molecular level.

**FIGURE 1:**
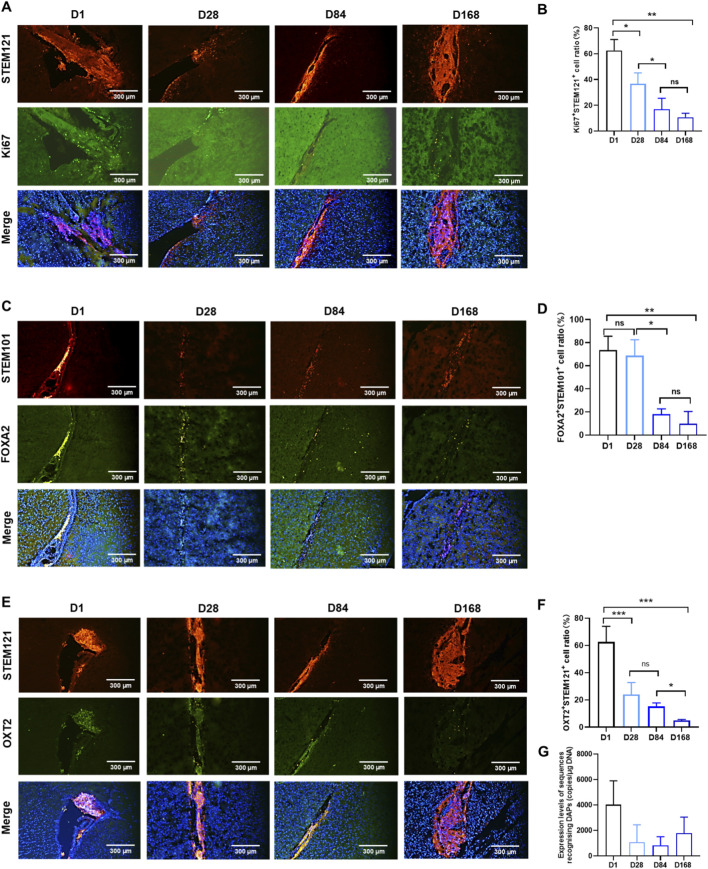
Expression levels of DAPs in the striatum region of mice after drug administration. **(A)** DAPs expressing both Ki61 and STEM121 were identified in the striatum at 1 day, 28 days, 84 days and 168 days post-administration. **(B)** The ratios of cells expressing both Ki67 and STEM121 are summarized (*P < 0.05, **P < 0.01). **(C)** DAPs expressing both FOXA2 and STEM101 were identified in the striatum at 1 day, 28 days, 84 days and 168 days post-administration. **(D)** The ratios of cells expressing both FOXA2 and STEM101 are summarized (*P < 0.05, **P < 0.01). **(E)** DAPs expressing both OXT2 and STEM121 were identified in the striatum at 1 day, 28 days, 84 days and 168 days post-administration. **(F)** The ratios of cells expressing both FOXT2 and STEM121 are summarized (*P < 0.05, **P < 0.001). **(G)** Expression levels of DNA sequences recognizing DAPs in the brains of mice at 1 day, 28 days, 84 days and 168 days are summarized.

Both Ki67-and STEM121-positive cells were identified at the administration site in the striatum of the animals in the low-dose group and high-dose group at 1 day (Ki67-positive cells were 62.85% ± 8.50%) post-administration, and these positive cells were distributed throughout the striatum region at 28 days and 84 days post-administration (Figures 1A,B). At 168 days post-administration, Ki67-and STEM121-positive cells were found in all the animals treated with DAP, and the percentage of remaining Ki67-positive cells was 10.71% ± 3.13%. In contrast, there are no Ki67-or STEM121-positive cells in peripheral tissues, such as the heart, liver, spleen, lungs, kidneys, spinal cord, testis, epididymis, ovary (in females), or blood. The expression rate of the DAP marker FOXA2 reached its peak at 1 day post-administration, at 73.43% ± 12.15%. Its expression rate slightly decreased by 28 days post-administration and significantly decreased to 9.63% ± 10.72% by 168 days post-administration (Figures 1C,D). The expression rate of the DAP marker OTX2 reached its peak by 1-day post-administration, at 62.58% ± 11.42%. Its expression rate slightly decreased thereafter and significantly decreased to 4.98% ± 0.73% by 168 days post-administration (Figures 1E,F).

Within 168 days post-administration of DAPs, human-specific sequences were detected only at the original drug administration site, and the target gene was absent in other peripheral organs and blood (Figure 1G). The copy number of the target gene in the brain tissue was the highest on the first day after the administration of DAPs, reaching 4,027.3 ± 1881.2 copies/μg DNA. It gradually decreased and reached the lowest value (845 ± 672.1 copies/μg DNA) by 84 days post-administration. By 168 days post-administration, the copy number of the target gene had increased to 1786.9 ± 1,263.4 copies/μg DNA. Therefore, the number of xenografted cells initially tended to decrease but then tended to increase *in vivo*.

### Differentiation of DAPs in immunodeficient mice

**FIGURE 2:**
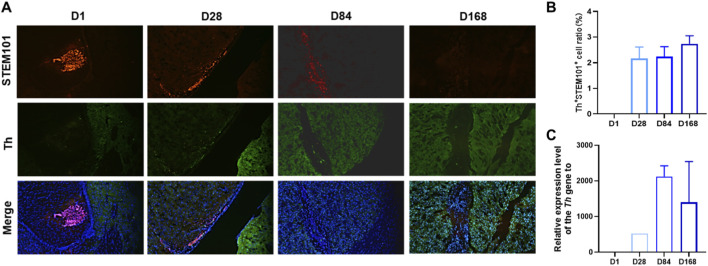
Expression levels of biomarkers for cell differentiation after the administration of DAPs in the striatum region of mice. **(A)** DAPs expressing both Th and STEM101 were identified in the striatum at 1 day, 28 days, 84 days and 168 days post-administration. **(B)** The ratios of cells expressing both Th and STEM101 are summarized. **(C)** Relative mRNA expression levels and *Th gene* sequences recognized by DAPs in the brains of mice at 1 day, 28 days, 84 days and 168 days are summarized.

The dopamine neuron differentiation markers Th and STEM101 were identified via immunohistochemistry from 1 day to 168 days after the administration of DAPs (Figures 2A,B). The expression level of Th was determined at 28 days post-administration, with an expression rate of 2.17% ± 0.45%. As time progressed, there was a slight increasing trend, and by 168 days post-administration, the expression rate reached 2.74% ± 0.31%.

The dopamine neuron differentiation marker gene *Th* was selected as the target mRNA for detection in brain tissue. No target RNA was detected 1 day after administration (Figure 2C). At 28 days post-administration, the test cells began to differentiate, and the target mRNA was detected in 1/6 of the samples, with a relative expression level of 524.6-fold. By 84 days post-administration, the differentiation rate increased, and the target mRNA was detected in 2/6 of the samples, with an increased degree of differentiation and a relative expression level of 2,123.6 ± 300.8 times. This result is consistent with the trend of enhanced dopaminergic differentiation in immunohistochemistry results (Figures 2A,B), further indicating that the differentiation of DAP cells may have been increased. By 184 days post-administration, the differentiation rate continued to increase, and the target mRNA was detected in 4/6 of the samples, with a slight decrease in the overall degree of differentiation and a relative expression level of 1,398.3 ± 1,145.3-fold.

### Single-dose toxicity study of DAPs in immunodeficient mice

**FIGURE 3:**
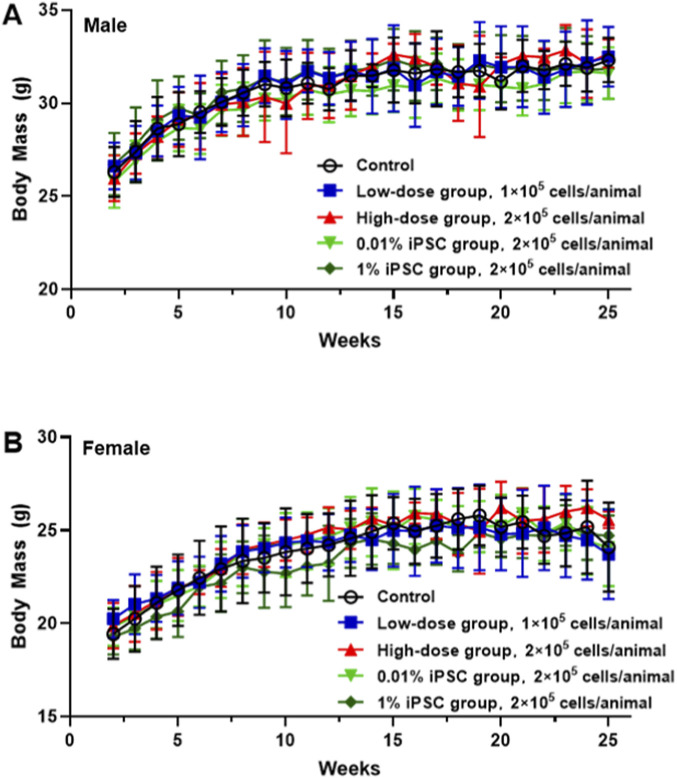
Changes in the body mass of the mice. The trends in the body masses of male **(A)** and female **(B)** mice 168 days after the administration of DAPs are shown.

There were no abnormal clinical symptoms in the animals that were administered DAPs, and the body mass (Figure 3) and food intake of the animals that were administered up to 2 × 10^5^ DAPs were not different from those of the control group.

**FIGURE 4:**
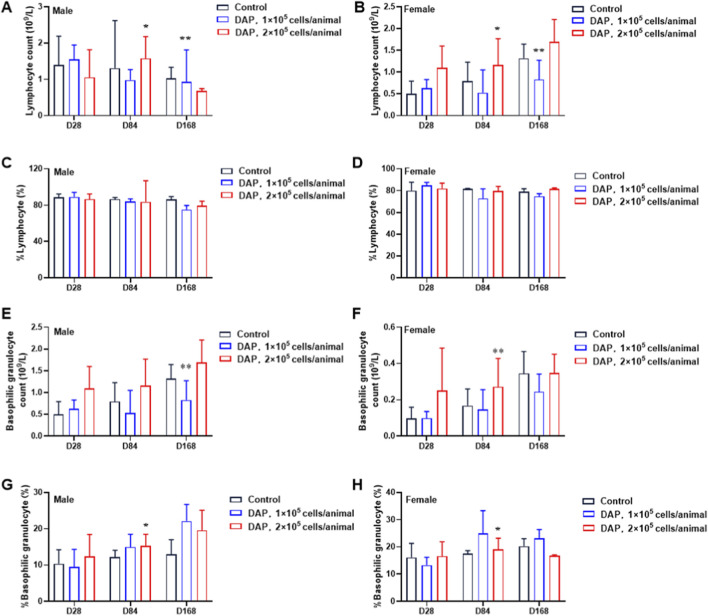
Changes in the hematological indices of DAPs in mice. The effects of DAPs on the lymphocyte count **(A,B)**, % lymphocyte count **(C,D)**, basophilic granulocyte count **(E,F)** and % basophilic granulocyte count **(G,H)** in both male and female mice at 28 days, 84 days and 168 days post-administration of DAPs are shown (compared with the control group on the same day, *P < 0.05, **P < 0.01).

The hematological data of the animals at 28 days, 84 days and 168 days post-administration are shown in Figure 4. By 84 days post-administration, compared with those in the solvent control group at the same time, the % lymphocyte density in female animals in the low-dose group was significantly lower (*P* < 0.05) than that in the solvent control group, whereas no statistically significant differences were observed in the other indicators. By 184 days post-administration, in male animals in the low-dose group, the %Lymph was significantly lower (*P* < 0.05, *P* < 0.01) and the %Baso was significantly greater (*P* < 0.05) than those in the solvent control group, while no significant difference was observed.

**FIGURE 5:**
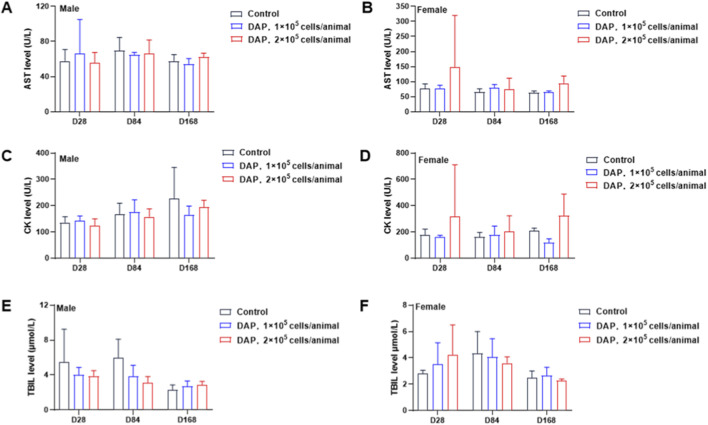
Changes in the serum biochemical indices of DAPs in mice. The effects of DAPs on the levels of AST **(A,B)**, CK **(C,D)** and TBIL **(E,F)** in both male and female mice at 28 days, 84 days and 168 days after the administration of DAPs are shown.

The serum biochemical data of the animals at 28 days, 84 days and 168 days post-administration are shown in Figure 5. By 84 days post-administration, compared with that of the solvent control group at the same time, the TBIL of male animals in the high-dose group was significantly lower (*P* < 0.05). By 184 days post-administration, compared with those in the solvent control group at the same time, the CK levels of female animals in the low-dose group were significantly lower (*P* < 0.05), and the AST levels of female animals in the high-dose group were significantly greater (*P* < 0.05). No statistically significant differences were observed in the other serum biochemical indicators.

**FIGURE 6:**
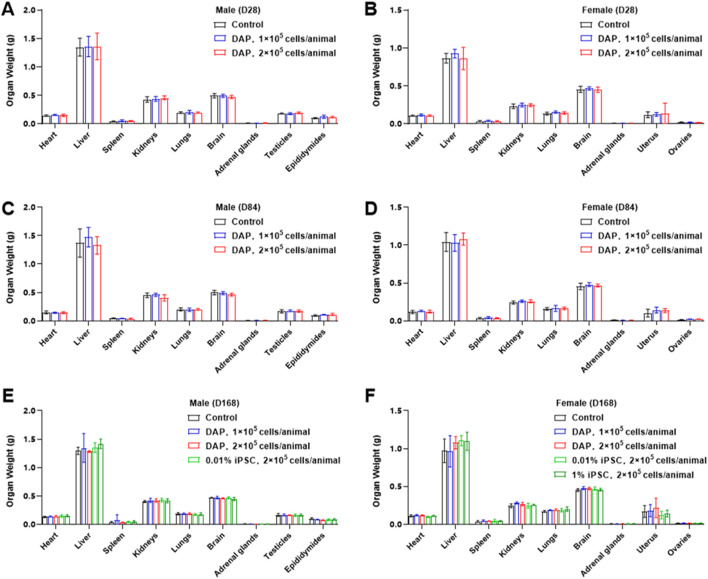
Changes in organ weights of DAPs in mice. The effects of DAPs on the weights of the heart, liver, spleen, kidneys, lungs, brain, adrenal glands, testicles (male only), epididymides (male only), uterus (female only) and ovaries (female only) at 28 days **(A,B)**, 84 days **(C,D)** and 168 days **(E,F)** after the administration of DAPs are shown.

Compared with those of the solvent control group, the absolute organ weights and organ coefficients of the adrenal glands, ovaries, hearts and livers of the animals (both male and female) in each dose group at different time points were not significantly different (Figure 6). By 184 days post-administration, the absolute weight and organ coefficient of the adrenal glands in male animals in the high-dose group were significantly decreased (*P* < 0.05). By 84 days post-administration, the absolute weight and organ coefficient of the ovaries in female animals in the low- and high-dose groups significantly increased (*P* < 0.05). The organ coefficient of the heart in female animals in the 0.01% iPSC group was significantly lower (*P* < 0.05), and by 184 days post-administration, the organ coefficient of the liver in male animals in the 1% iPSC group was significantly greater (*P* < 0.05). However, no corresponding histopathological changes were observed for any of these changes, and no significant differences were found in the remaining groups. Therefore, these changes are not related to the administration of the test substance. No significant differences were detected in the absolute organ weights or organ coefficients of the remaining groups of animals in each tissue or organ.

**FIGURE 7:**
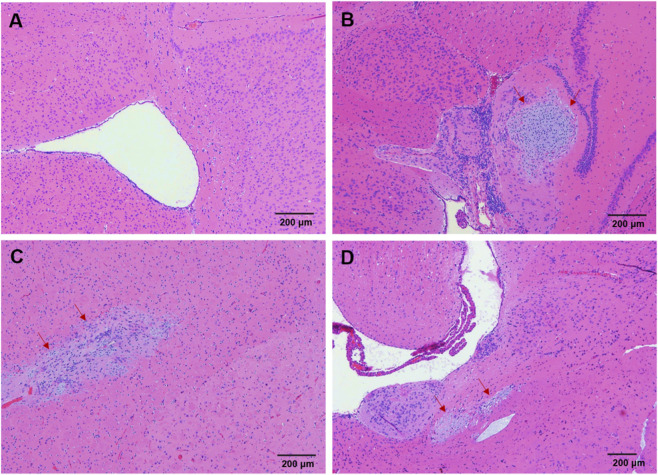
Pathological changes in cerebral tissue. **(A)** Normal brain. **(B)** Mild cell aggregation around the ventricles and meninges. **(C)** Slight cell aggregation in the striatum. **(D)** Mild cell aggregation in the striatum and around the ventricles. Regions with aggregated cells are labeled with red arrow.

The main histopathological change related to the test substance was aggregation in the brain (Figure 7). By 84 days post-administration, cellular aggregations had occurred in the animals given DAPs, with 1/5 of the male animals and 2/5 of the female animals in the low-dose group and 1/4 of the male animals in the high-dose group affected. The lesion severity was mild. The cellular aggregations were in the periventricular area and/or around the striatum and were related to the administration of the test substance. By 184 days post-administration, cellular aggregations occurred in the animals given DAPs and those given iPSCs, with mild to moderate severity, focal or multifocal distribution, and were located in the periventricular, striatal and/or meningeal regions. The affected animals included 2/5 male animals in the low-dose group, 1/3 female animals in the high-dose group, 1/6 male animals and 3/4 female animals in the 0.01% iPSC group, and 4/6 male animals and 1/5 female animals in the 1% iPSC group. Cell aggregation might be induced by either test cells or the glial cell response stimulated by the test cells. When the cellular aggregations were in the striatum or white matter of the brain, there were mucoid matrix changes. The animals in the 0.01% iPSC group and the 1% iPSC group were given the same dose of DAP as the high-dose group and were also given 0.01% iPSCs and 1% iPSCs, respectively. Therefore, this lesion was related to the administration of the test cells and iPSCs. All the other microscopic observations revealed the expected lesion types and incidence rates in B-NDG mice, and the incidence rates were similar across all the test groups; thus, typical spontaneous or incidental lesions in B-NDG mice are considered common.

## Discussion

Traditional available therapeutic approaches fail to effectively regenerate dopaminergic neurons or halt the progression of neuronal degeneration and thus cannot fundamentally alter the disease course of PD (Kirkeby et al., 2023; Jeffr et al., 2022). Recently, hiPSCs have demonstrated advantages in the treatment of PD (Morizane, 2023; Brot et al., 2022). Jeon et al. reported promising effectiveness data of hiPSC-derived midbrain dopaminergic cells for PD patients, and no adverse effects were observed in a 9-month NSG mouse study (Jeon et al., 2025; Park et al., 2024). Park et al. generated highly purified human embryonic stem cell-derived midbrain dopaminergic cells, and improved behavior was observed in immunodeficient rats treated with 5,000–10,000 cells (Park et al., 2024). Nevertheless, post-transplantation risk, specific ectopic distribution and tumorgenicity remain the primary concerns (Morizane, 2023; Sahana et al., 2023). There is currently a lack of comprehensive preclinical safety evaluation strategies for hiPSCs. DAPs are high-purity subtype-specialized dopaminergic neural precursor cells; they exert their therapeutic effects on PD mainly by forming local neural circuits with host neurons through cell transplantation and reconstructing functional synaptic connections (Valadez-Barba et al., 2023). This study, which was conducted under good laboratory conditions, provided long-term biodistribution and safety data of DAPs in NSG mice and supported phase I clinical trials in China and the United States.

To use iPSC technology for treating PD, a sufficient number of mDA neurons must survive after transplantation. A multidimensional evaluation approach for assessing the distribution and differentiation of DAPs *in vivo* was established by combining immunofluorescence staining and qPCR for the first time in this study. It comprehensively analyzes the situation of transplanted test cells at the cellular and genetic levels, and displays the good colonization and survival of test cells in the brain at different levels. It also visualizes the gradual differentiation process of test cells after prolonged drug administration. This evaluation method could not only be applied to DAPs, but also provide a powerful tool for preclinical safety assessment of other types of regenerative medicine products in the future.

As demonstrated, STEM121 expression was detected in the striatum on the administration side in 17/18 animals at 1-, 28- and 84-days post-administration of DAPs; at 6 months after administration, STEM121-positive cells were detected in the hippocampus in 1/5 animals and in the striatum in 4/5 animals at 6 months post-administration of DAPs; however, STEM121-positive cells were not detected in the cerebellum, blood or peripheral organs. Our qPCR results revealed that human cell-specific sequences for recognizing DAPs were detected only in the brain but not in the blood or peripheral organs. As such, subsequent to the administration, the DAPs were distributed only to the adjacent tissues near the administration site. As the *in vivo* proliferation period increased, DAP migration to the hippocampus was observed in few animals at 6 months post-administration. The PCR data also revealed that the gene copy number of the DAPs peaked at 1-day post-administration, after which it tended to first increase but then decrease. As the target gene was designed for human-specific sequences, the degree of differentiation of DAPs affects its quantitative data. Therefore, the number of DAPs peaked at 1-day post-administration, then decreased gradually to a stable level, and the DAPs persisted in the mice for at least 6 M. However, as the degree of differentiation of the test cells increased, the cell number increased.

In this study, an immunofluorescence method was used to assess *in vivo* cell differentiation. As OCT4-positive cells recognizing hiPSCs were not found at 1-, 28-, 84- and 168-days post-administration, the DAPs did not contain hiPSCs, and no hiPSCs were generated after transplantation. In contrast, the expression levels of DAP markers (including Ki67, FOXA2, OTX2, STEM101 and STEM121) markedly increased at 1-day post-administration and then gradually decreased, and only a small amount was detected at 168 days post-administration. The qPCR results indicated that the target RNA (TH) for differentiated cells was not absent at 1-day post-administration, but it gradually increased from 28–168 days post-administration and peaked at 84 days post-administration. In summary, DAP cells gradually differentiated into neuronal cells, and the differentiation rate gradually increased over time. Daisuke Doi et al. administered DAPs derived from iPSCs into the right striatum of immunodeficient NOG mice, and immunohistochemical staining revealed that the transplanted human cells (KU80+) were found only in the brain but not in other organs (Daisuke et al., 2020). The preclinical data on Parkinson’s disease released by the Harvard team further suggested that mesencephalic dopaminergic cells differentiated from iPSCs exhibited good safety in mouse models (Jeon et al., 2025). Their biodistribution data demonstrated that mesencephalic dopaminergic cells differentiated from iPSCs could be found only in brain tissue by 39 weeks post-administration. In addition, the volume of the grafted cells increased approximately tenfold, whereas the cell density decreased, indicating cell differentiation and synaptic extension. Although TH is the primary biomarker for dopaminergic neurons in this study, we acknowledge that transcriptional regulation can also affect the elevation of TH mRNA levels. However, based on the immunohistochemical results of this study and the literature report by Jeon et al. (2025), we believe that this is more likely to indicate an increase in differentiation degree. In future research, we will conduct a comprehensive evaluation using multiple biomarkers including NURR1, LMX1A, and DAT to further confirm this conclusion. This evidence is consistent with our findings, suggesting that DAPs do not spread systemically after transplantation and are completely localized to the targeted brain region, which meets the expected clinical local treatment characteristics of DAPs.

The distribution and persistence of cell therapy products in the body are crucial factors affecting their efficacy and safety. Therefore, during the nonclinical research stage, the biological distribution characteristics of stem cells, such as the migration, colonization, differentiation, and persistence of differentiated cells, undifferentiated cells, and expected therapeutic cells in the recipient, should be dynamically observed. In addition, combined immunofluorescence and qPCR approaches are used to investigate the biodistribution of DAPs (Jinghua et al., 2021). Immunofluorescence staining was applied to identify the differentiation marker protein STEM121 of DAPs, enabling visual monitoring of the distribution and differentiation of DAPs in the brain and body. However, the sensitivity of immunofluorescence staining is relatively poor, which may lead to high background and false positive results, and it is difficult to perform quantitative analysis. The use of qPCR in conjunction can accurately and comprehensively detect peripheral organs and reflect the relative changes in the number of DAPs after transplantation. Moreover, the limitations of the above approaches cannot be neglected. For example, neither method can achieve continuous monitoring of the same animal, and the degree of differentiation of DAPs may be limited due to differences in the microenvironment between normal and diseased animals. Hence, more specific, stable and efficient methods need to be further developed to achieve comprehensive and dynamic observations of the *in vivo* distribution of stem cell products.

In this study, DAPs were administered to the unilateral striatum of B-NDG mice via a single stereotactic injection. DAPs at doses of 1 × 10^5^ cells/animal and 2 × 10^5^ cells/animal did not affect the mortality, body mass or food intake of the mice. Some adverse intergroup changes in hematological and blood biochemical indicators were observed, as were histopathological examinations. The overall lymphocyte percentage was slightly lower in the females in the low-dose group at 168 days post-administration, the overall percentage was slightly lower, and the percentage of BASO was slightly higher in the males in the low-dose group at 84 days. These changes were observed only in the low-dose group, with no significant dose-dependent changes observed. The combination of normal results with other indicators was considered unrelated to the test substance. The serum biochemical data revealed that TBIL was slightly low in the males of the high-dose group by 84 days post-administration; CK values decreased in the females of the low-dose group by 168 days post-administration, and no significant changes were observed in the high-dose group during the same period; AST values increased in the females of the high-dose group. The above changes were all considered individual deviations. These fluctuations were possibly due to the stress response occurring in immunocompromised animals. On the basis of previous preclinical studies, transplantation surgery and the inherent characteristics of immunodeficient animals often lead to multiorgan lymphatic tissue cell inflammation, which results in animal injury or even death. Drawing from previous research experience and our data, changes in hematological and blood biochemical indicators may not be associated with DAPs. Therefore, it could be concluded that the DAPs did not cause significant toxicity in mice and exhibited good preclinical safety.

In animals administered DAPs and iPSCs, mild mixed cell aggregation was observed in the brain, which is associated with the exogenous implantation caused by DAPs or iPSCs. Since no neuronal damage was observed in any of the affected animals, cell aggregation without adverse effect. In addition, no abnormal proliferation or tumor formation was observed in any tissue of the animals that were administered DAPs or iPSCs.

The tumorigenicity risk of DAPs stems primarily from residual undifferentiated iPSCs, as well as potential genomic instability or the introduction of other transformed cell components during the reprogramming and *in vitro* culture expansion stages (Sato et al., 2019). Undifferentiated iPSCs possess inherent tumorigenicity and may induce teratomas and genetic mutations (Sonntag et al., 2018). Therefore, this study investigated whether residual undifferentiated iPSCs could form tumors through *in situ* drug administration, assessing the potential tumorigenicity of undifferentiated iPSC residues. Our data revealed no abnormal proliferation or tumorigenesis in various tissues of the low- and high-dose groups or in the 0.01% and 1% iPSC groups.

Owing to the difference between the healthy mouse model and the human cell genome, NSG mice cannot adequately simulate the microenvironment of humans to mimic the *in vivo* process of DAPs. Moreover, nerve cells can take years or even decades to proliferate and differentiate in humans after administration. In this study, we observed NSG mice for 6 months after administration on the basis of the lifespan of the animals; however, the results might still be insufficient to assess the long-term risk of DAPs *in vivo*. To mimic clinical situations in animal models, the toxicological risk of iPSCs generally needs to be monitored until at least 6–10 months after transplantation (Brot et al., 2022). The safety and effectiveness of clinical-grade human induced pluripotent stem cell (hiPSC)-derived mesencephalic dopaminergic cells were previously investigated by transplanting DAPs into NSG mice, and no tumor formation over a 39-week period was found (Jeon et al., 2025). The limitations of animal survival and observation periods need to be overcome, and animal models that are more similar to the human microenvironment could be used in future studies. This could be further validated in clinical studies for a more comprehensive evaluation of the safety and effectiveness of iPSC-derived DAPs. In addition, we shall use human derived cell markers (e.g., STEM121), astrocytes markers (e.g., GFAP), and microglia markers (e.g., Iba1) for immunofluorescence co-staining to further investigate the cellular origin of aggregates.

## Conclusion

Improving safety evaluation strategies requires efficient approaches to overcome the barriers between innovative discovery and clinical translation of hiPSCs. This study established a novel approach for determining biodistribution and differentiation *in vivo*, provided a safety evaluation strategy for iPSC-derived DAPs and demonstrated safety in mice. We pioneered the introduction of the striatum drug delivery technique in immunodeficient mice in a study performed under GLP conditions. The clinical translation of DAPs and their phase I clinical trials in China and the United States are supported by the fundamental safety data provided by these data.

## Data Availability

The raw data supporting the conclusions of this article will be made available by the authors, without undue reservation.

## References

[B1] Brot S. Thamrin N. P. Bonnet M. L. Francheteau M. Patrigeon M. Belnoue L. (2022). Long-term evaluation of intranigral transplantation of Human iPSC-Derived dopamine neurons in a Parkinson's Disease mouse model. Cells 11 (10), 1596. 10.3390/cells11101596 35626637 PMC9140181

[B2] Cha Y. Park T. Y. Leblanc P. Kim K. S. (2023). Current status and future perspectives on stem cell-based therapies for Parkinson's Disease. J. Mov. Disord. 16 (1), 22–41. 10.14802/jmd.22141 36628428 PMC9978267

[B3] Daisuke D. Hiroaki M. Tetsuhiro K. Ikeda M. Hiramatsu S. Yoshida K. (2020). Preclinical study of induced pluripotent stem cell-derived dopaminergic progenitor cells for Parkinson's disease. Nat. Communications 11 (1), 3369. 10.1038/s41467-020-17165-w PMC7338530 32632153

[B4] Hatou S. Sayano T. Higa K. Inagaki E. Okano Y. Sato Y. (2021). Transplantation of iPSC-derived corneal endothelial substitutes in a monkey corneal edema model. Stem Cell Res. 55, 102497. 10.1016/j.scr.2021.102497 34411973

[B5] Jeffrey K. Oh T. Daadi E. S. Daadi M. M. Thomas O. (2022). Human induced pluripotent stem cell phenotyping and preclinical modeling of familial parkinson’s disease. Genes 13 (11), 1937. 10.3390/genes13111937 36360174 PMC9689743

[B6] Jeon J. Cha Y. Hong Y. J. Lee I. H. Jang H. Ko S. (2025). Preclinical safety and efficacy of human induced pluripotent stem cell-derived products for autologous cell therapy in Parkinson's disease. Cell Stem Cell 32 (3), 343–360.e7. 10.1016/j.stem.2025.01.006 39952239 PMC11980241

[B7] Jinghua P. Susan Z. Dubose B. N. Hill E. J. Navare M. Claros N. (2021). Preclinical efficacy and safety of a human embryonic stem cell-derived midbrain dopamine progenitor product, MSK-DA01. Cell Stem Cell 28 (2), 217–229. 10.1016/j.stem.2021.01.004 33545080 PMC7903922

[B8] Kirkeby A. Nelander J. Hoban D. B. Rogelius N. Bjartmarz H. Novo Nordisk Cell Therapy R&D (2023). Preclinical quality, safety, and efficacy of a human embryonic stem cell-derived product for the treatment of Parkinson's disease, STEM-PD. Cell Stem Cell 30(10), 1299–1314. 10.1016/j.stem.2023.08.014 37802036

[B9] Liu Z. Cheung H. H. (2020). Stem cell-based therapies for parkinson disease. Int. J. Mol. Sci. 21(21), 8060. 10.3390/ijms21218060 33137927 PMC7663462

[B10] Mehler J. V. Burns J. C. Stauss H. Francis R. J. Moore M. L. (2020). Human iPSC-Derived neural crest stem cells exhibit low immunogenicity. Mol. Ther. - Methods and Clin. Dev. 16, 161–171. 10.1016/j.omtm.2019.12.015 32055644 PMC7005462

[B11] Mominur M. R. Rezaul M. I. Touhidul M. I. Harun-Or-Rashid M. Islam M. Abdullah S. (2022). Stem cell transplantation therapy and neurological disorders: current status and future perspectives. Biology 11(1), 147. 10.3390/biology11010147 35053145 PMC8772847

[B12] Morizane A. (2023). Cell therapy for Parkinson's disease with induced pluripotent stem cells. Inflamm. Regen. 43 (1), 16. 10.1186/s41232-023-00269-3 36843101 PMC9969678

[B13] Myriam L. Juliane P. Leon P. Zogg M. Thiruchelvam S. Müller M. (2023). Identification of marker genes to monitor residual iPSCs in iPSC-derived products. Cytotherapy 25 (1), 59–67. 10.1016/j.jcyt.2022.09.010 36319564

[B14] Nakamura R. Nonaka R. Oyama G. Jo T. Kamo H. Nuermaimaiti M. (2023). A defined method for differentiating human iPSCs into midbrain dopaminergic progenitors that safely restore motor deficits in Parkinson's disease. Front. Neurosci. 17, 1202027. 10.3389/fnins.2023.1202027 37502682 PMC10368972

[B15] Park I. H. Zhao R. West J. A. Yabuuchi A. Huo H. Ince T. A. (2008). Reprogramming of human somatic cells to pluripotency with defined factors. Nature 451:141–146. 10.1038/nature06534 18157115

[B16] Park S. Park C. W. Eom J. H. Jo M. Y. Hur H. J. Choi S. K. (2024). Preclinical and dose-ranging assessment of hESC-derived dopaminergic progenitors for a clinical trial on Parkinson's disease. Cell Stem Cell 31 (1), 25–38. e8. 10.1016/j.stem.2023.11.009 38086390

[B17] Pires A. O. Teixeira F. G. Mendes-Pinheiro B. Serra S. C. Sousa N. Salgado A. J. (2017). Old and new challenges in Parkinson's disease therapeutics. Prog. Neurobiol. 156, 69–89. 10.1016/j.pneurobio.2017.04.006 28457671

[B18] Sahana B. S. Haritha D. Jillian B. Switalski S. Shafa M. Panchalingam K. M. (2023). Characterization of human induced pluripotent stems cells: current approaches, challenges, and future solutions. Biotechnol. Rep. 37, e00784. 10.1016/j.btre.2023.e00784 PMC9929203 36818379

[B19] Sato Y. Bando H. Di Piazza M. Gowing G. Herberts C. Jackman S. (2019). Tumorigenicity assessment of cell therapy products: the need for global consensus and points to consider. Cytotherapy 21 (11), 1095–1111. 10.1016/j.jcyt.2019.10.001 31711733

[B20] Sequiera G. L. Srivastava A. Sareen N. Yan W. Alagarsamy K. N. Verma E. (2022). Development of iPSC-based clinical trial selection platform for patients with ultrarare diseases. Sci. Adv. 8 (14), eabl4370. 10.1126/sciadv.abl4370 35394834 PMC8993122

[B21] Sonntag K. C. Song B. Lee N. Jung J. H. Cha Y. Leblanc P. (2018). Pluripotent stem cell-based therapy for Parkinson's disease: current status and future prospects. Prog. Neurobiol. 168, 1–20. 10.1016/j.pneurobio.2018.04.005 29653250 PMC6077089

[B22] Stoddard-Bennett T. Pera R. R. (2020). Stem cell therapy for Parkinson's disease: safety and modeling. Neural Regen. Res. 15(1), 36–40. 10.4103/1673-5374.264446 31535640 PMC6862409

[B23] Stoddard-Bennett T. Reijo Pera R. (2019). Treatment of Parkinson's disease through personalized medicine and induced pluripotent stem cells. Cells 8(1), 26. 10.3390/cells8010026 30621042 PMC6357081

[B24] Takahashi K. Tanabe K. Ohnuki M. Narita M. Ichisaka T. Tomoda K. (2007). Induction of pluripotent stem cells from adult human fibroblasts by defined factors. Cell. ;131(5):861–872. 10.1016/j.cell.2007.11.019 18035408

[B25] Valadez-Barba V. Juárez-Navarro K. Padilla-Camberos E. Díaz N. F. Guerra-Mora J. R. Díaz-Martínez N. E. (2023). Parkinson's disease: an update on preclinical studies of induced pluripotent stem cells. Neurol. 38 (9), 681–694. 10.1016/j.nrleng.2023.10.004 37858889

[B26] Zhao T. Zhang Z. N. Westenskow P. D. Todorova D. Hu Z. Lin T. (2015). Humanized mice reveal differential immunogenicity of cells derived from autologous induced pluripotent stem cells. Cell Stem Cell 17 (3), 353–359. 10.1016/j.stem.2015.07.021 26299572 PMC9721102

